# Dengue, chikungunya and Zika virus infections among Dutch travellers to Suriname: a prospective study during the introduction of chikungunya and Zika virus, 2014 to 2017

**DOI:** 10.2807/1560-7917.ES.2023.28.2.2200344

**Published:** 2023-01-12

**Authors:** Femke W Overbosch, Janke Schinkel, Amy Matser, Gerrit Koen, Irene Prange, Maria Prins, Gerard JB Sonder

**Affiliations:** 1 Department of Infectious Diseases, Public Health Service (GGD), Amsterdam, the Netherlands; 2 Department of Medical Microbiology, Laboratory of Clinical Virology, Amsterdam University Medical Centers, location Academic Medical Center, Amsterdam, the Netherlands; 3 Department of Infectious Diseases Research and Prevention, Public Health Service (GGD), Amsterdam, the Netherlands; 4 Department of Internal Medicine, Amsterdam Infection and Immunity Institute (AIII), Amsterdam UMC, location Academic Medical Center, Amsterdam, the Netherlands

**Keywords:** travellers, Suriname, dengue virus infections, chikungunya virus infections, Zika virus infections, prospective

## Abstract

**Background:**

Suriname, a country endemic for dengue virus (DENV), is a popular destination for Dutch travellers visiting friends and relatives and tourist travellers. Chikungunya and Zika virus (CHIKV, ZIKV) were introduced in 2014 and 2015, respectively. Data on infection risks among travellers are limited.

**Aim:**

We aimed to prospectively study incidence rate (IR) and determinants for DENV, ZIKV and CHIKV infection in adult travellers to Suriname from 2014 through 2017.

**Methods:**

Participants kept a travel diary and were tested for anti-DENV, anti-ZIKV and anti-CHIKV IgG antibodies (Euroimmun). Selected samples were subjected to an in-house DENV and ZIKV PRNT50. The IR (infections/1,000 person-months of travel) and IR ratio and determinants for infection were calculated.

**Results:**

Travel-acquired infections were found in 21 of 481 participants: 18 DENV, four ZIKV and two CHIKV, yielding an IR_DENV_ of 47.0 (95% CI: 29.6–74.6), IR_ZIKV_ of 11.6 (95% CI: 4.4–31.0) and IR_CHIKV_ of 5.6 (95% CI: 1.4–22.2)/1,000 person-months. In nine DENV and three ZIKV infected participants, infections were PRNT50-confirmed, yielding a lower IR_DENV_ of 23.3 (95% CI: 12.1–44.8) and an IR_ZIKV_ of 8.4 (95% CI: 2.7–26.1) per 1,000 person-months. Tourist travel was associated with DENV infection. ZIKV and CHIKV infections occurred soon after their reported introductions.

**Conclusions:**

Despite an overestimation of serologically confirmed infections, Dutch travellers to Suriname, especially tourists, are at substantial risk of DENV infection. As expected, the risk of contracting ZIKV and CHIKV was highest during outbreaks. Cross-reaction and potential cross-protection of anti-DENV and -ZIKV antibodies should be further explored.

**Key public health message**

**What did you want to address in this study?**

Travellers to Suriname can acquire dengue, and since 2014/2015 Zika and chikungunya as well. We wished to know the risks and risk factors of these three infections among travellers visiting friends and relatives and tourist travellers from the Netherlands.

**What have we learnt from this study?**

Dutch travellers run a substantial risk of dengue, especially the tourist travellers. We also found that participants who travelled during outbreak periods had a considerable risk to contract Zika or chikungunya.

**What are the implications of your findings for public health?**

The European areas where the mosquitoes live that can transmit dengue, Zika and chikungunya virus have expanded and are at risk for virus introduction by ill travellers, especially in summer months. Knowledge about the risk of dengue, Zika and chikungunya among travellers to popular endemic destinations informs targeted travel health advice to people most at risk, and can therefore reduce the risk of virus introduction in Europe.

## Introduction

Several countries in Europe have close ties with specific tropical or subtropical countries, and travellers from connected European areas visit these (sub)tropical countries frequently. Suriname for example, a dengue-endemic country in South America with 575,763 inhabitants (2016) and a former colony of the Netherlands that gained its independence in 1975, is one of the most popular destinations among Dutch travellers to tropical and subtropical countries [1-3].

Dengue is a mosquito-borne viral disease with a clinical spectrum ranging from asymptomatic or mild influenza-like to severe disease, including death. Identification of risk groups for infection with dengue virus (DENV) during travel – both for primary and secondary infections – is important, as risk groups will benefit most from tailored prevention strategies including future vaccines. This is challenging, however, as retrospective research on travellers returning with illness underestimates incidence rates because it overlooks asymptomatic and mild infections. Also, clinical misdiagnosis can occur in countries endemic for Zika and chikungunya viruses (ZIKV and CHIKV), as the clinical spectrum of these mosquito-borne viruses may resemble dengue [4,5]. Finally, cross-reactivity of antibodies against flaviviruses such as DENV, ZIKV, yellow fever virus (YFV), tick-borne encephalitis virus (TBEV) and Japanese encephalitis virus (JEV) can occur and complicate interpretation of serological results [6,7].

So far, no cure exists, nor is a vaccine for travellers available [8]. Avoiding mosquito bites is currently the most appropriate prevention strategy. In addition, secondary DENV infection with a heterotypic serotype (DENV_1, 2, 3 or 4_), can be more severe, but tertiary DENV infections are rarely seen [6]. Presence of pre-existing DENV antibodies is therefore not conclusive for immunity, nor for severe disease. A previous study found pre-existing DENV antibodies among 81% of Surinamese migrants in Amsterdam [9]. Migrant travellers visiting friends and relatives (VFR) are known to be at increased risk of travel-related diseases in their country of origin, such as malaria, hepatitis A and typhoid fever, and might similarly be at increased risk for secondary DENV infection [10-12]. Identifying determinants for DENV infections among European travellers to tropical or subtropical countries such as Suriname will therefore be important.

To estimate the risk and determinants of travel-acquired DENV infection, we conducted a prospective study among VFR and tourist travellers to Suriname. As ZIKV and CHIKV were introduced on the American continent during the study period, we decided to expand the study with ZIKV and CHIKV infections.

## Methods

The aim was to prospectively study the attack rate (AR), incidence rate (IR) and determinants of travel-acquired DENV, ZIKV and CHIKV infections among Dutch travellers visiting friends and relatives and tourist travellers to Suriname.

### Study population

Travellers to Suriname seeking pre-travel services at the Public Health Service (GGD) of Amsterdam from March 2014 through October 2017 were eligible to participate if born in Suriname (defined as VFR) or the Netherlands (defined as tourist). Inclusion criteria were age ≥ 18 years and intended travel duration of ≤ 3 months. All participants were seen by a medical doctor or nurse specialising in travel medicine and were advised according to Dutch National Guidelines on Traveller’s Health Advice [13], receiving oral and written information about avoiding mosquito bites.

### Study procedures

Before departure, a standardised questionnaire in Dutch was used to collect each traveller’s socio-demographic data and vaccination history. Participants were given a digital thermometer (Domotherm Rapid 30 s, UEBE Medical GmbH, Wertheim, Germany) and asked to take their temperature if they felt feverish and also to keep a structured, daily paper travel diary until 2 weeks after return, recording their itinerary (in Suriname, the capital Paramaribo, coastal areas or inland; the Netherlands; other countries), their use of N,N-diethyl-meta-toluamide (DEET), presence of symptoms (fever if ≥ 38 °C, myalgia, arthralgia, headache, retro-orbital pain, diarrhoea, rash or other) and physician visits. An English translation of the full questionnaire is provided in the Supplement (part 1). After their return, the travellers were to present to the health service once more and their diaries were checked for clarity and completeness and, if necessary, complemented by a specialised nurse or physician together with the participant. The diaries were subsequently entered in a digital database using single data entry, in which unticked boxes – indicating absence of symptoms or treatment – were considered the default setting. Participants donated a blood sample pre-travel and during their return visit 2–4 weeks after return.

### Laboratory methods

**Figure 1:**
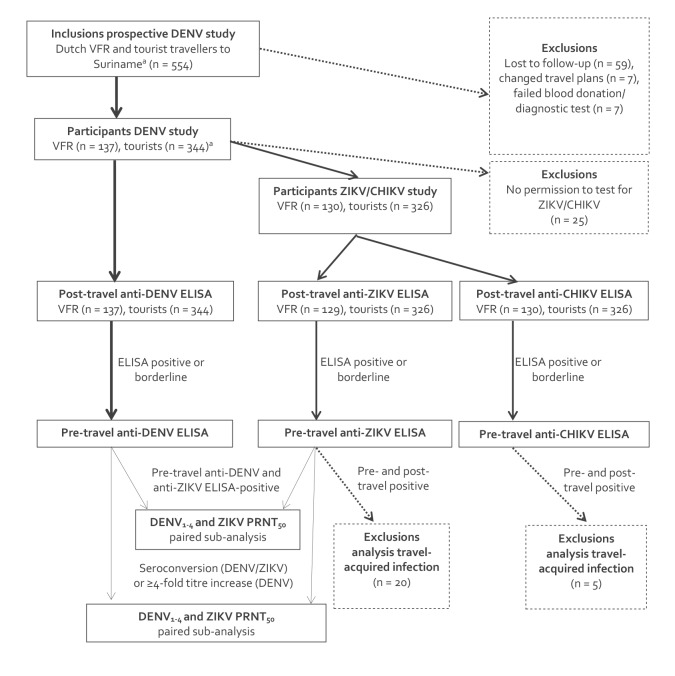
Prospective study among Dutch travellers to Suriname on travel-acquired dengue, Zika and chikungunya virus infections, the Netherlands, 2014–2017 (n = 554)

CHIKV: chikungunya virus; DENV: dengue virus; ELISA: enzyme-linked immunosorbent assay; PRNT: plaque-reduction neutralisation test; VFR: travellers visiting friends and relatives; ZIKV: Zika virus.

^a^ 14 participants who donated their post-travel sample < 14 days after travel (range 9–13 days) were not excluded, as we assumed very limited bias by including them. Tourist travellers include 65 second-generation migrants.

All blood samples were centrifuged, and serum samples were frozen at −80 °C within 24 h after blood donation. After all participants had returned, post-travel samples were serologically tested for immunoglobulin (Ig)G antibodies against DENV antigen serotypes 1, 2, 3 and 4. If the participants had consented, post-travel samples were also tested for anti-ZIKV IgG and anti-CHIKV IgG. Pre-travel samples were only tested in participants with a positive or borderline post-travel test result (Figure 1). Anti-DENV enzyme-linked immunosorbent assay (ELISA) IgG, anti-ZIKV ELISA IgG and anti-CHIKV ELISA IgG tests were used according to manufacturer’s instructions (Euroimmun, Lübeck, Germany). Reported sensitivity and specificity were, respectively, 98–100% and 100% for DENV, 100% and 97% for ZIKV, and 88% and 95% for CHIKV [14-16]. Paired sera were tested in participants with a positive or borderline post-travel test result (Figure 1). The presence of anti-DENV, anti-ZIKV or anti-CHIKV IgG in the pre-travel sample was considered suggestive of a previous infection. Seroconversion between paired sera was considered suggestive of a travel-acquired infection. An at least fourfold post- to pre-travel ratio of anti-DENV IgG was considered suggestive of a secondary travel-acquired DENV infection.

To examine for possible cross-reactivity in the ELISAs, for budgetary reasons, we subjected a selection of samples to an in-house (Amsterdam University Medical Centers) 50% plaque-reduction neutralisation test (PRNT50) against DENV serotypes 1–4 and ZIKV [17]. The PRNT is a serological test in which antibodies neutralise virus by preventing the virus to form plaques in a cell monolayer. We compared plaque formation after addition of a participant sample (in which the antibodies of interest might be present) vs a control without an added sample. The PRNT50 laboratory procedures are described in the Supplement (part 2). Samples were selected for PRNT50 if (i) the pre-travel anti-DENV and anti-ZIKV ELISA yielded a positive test result or (ii) a travel-acquired DENV and/or ZIKV was found in paired ELISA results. The PRNT50 was considered (i) a confirmation of seropositivity for DENV and/or ZIKV if the pre-travel serum inhibited plaque formation by ≥ 50% compared with sample-free controls and (ii) a confirmation of seroconversion if in paired sera the PRNT50 result converted from < 50% to ≥ 50%, or a confirmed secondary DENV infection if the pre-travel PRNT50 result was ≥ 50% for one serotype combined with a PRNT50 result conversion in paired sera from < 50% to ≥ 50% for at least one other serotype.

### Definitions

To examine a potential association due to cross-reaction between flavivirus vaccines and serological test results, we defined ‘pre-travel vaccination status’ as the total number of all flavivirus vaccinations against yellow fever (YF), Japanese encephalitis (JE) and tick-borne encephalitis (TBE) received before pre-travel blood donation (for example for previous travel), registered as in the participants’ international certificate of vaccination and/or the public health service patient file system and/or self-reported by the participant. As pre-travel blood donation was incorporated in the pre-travel health consultation for a trip to Suriname, which potentially included vaccination against YF, the ‘post-travel vaccination status’ was defined as the total number of all additional YF, JE and TBE vaccinations received during or after the pre-travel blood donation. Use of DEET was quantified by dividing the reported number of days of DEET usage by the number of travel days.

### Statistical analysis

#### Travel-acquired infections

We used Poisson regression analysis to examine the association between selected variables and travel-acquired infections. Participants having anti-ZIKV or anti-CHIKV IgG in the pre-travel sample were considered immune and excluded from the analysis of travel-acquired ZIKV or CHIKV infection. Participants having anti-DENV IgG in the pre-travel sample were considered at risk for secondary DENV infections and therefore included in the analysis of travel-acquired DENV infections. For individuals infected during travel, the moment of infection was estimated as the midpoint between their arrival and departure dates.

We calculated attack rates (AR, the number of participants with a travel-acquired infection divided by total number of participants, expressed as percentage), incidence rates (IR, expressed as the number of infections acquired during 1,000 person-months (pm) of travel), and incidence rate ratios (IRR) of infections based on serology suggestive of travel-acquired infection for the entire cohort, independent of when ZIKV and CHIKV were introduced into Suriname. For each disease under study, we assessed the association with sex, age group, country of birth, vaccination status, usage of DEET and calendar year of travel. Variables with a p value < 0.1 in univariable analysis were included in the multivariable model and retrained in the final model using a backwards stepwise approach.

In a sensitivity analysis, we repeated the Poisson analysis for the PRNT50-confirmed travel-acquired DENV and ZIKV infections. Finally, we estimated IR for travel-acquired ZIKV and CHIKV infections based on serology and on PRNT50 during the outbreak periods, limiting the exposure time to the estimated duration of the outbreaks. For ZIKV, duration was 1 September 2015 through 25 April 2016 (first case reported on 2 October 2015); for CHIKV, duration was 1 June 2014 (also date of first case reported in Suriname) through 9 March 2015 [4,5,18,19].

#### Pre-travel infections

To determine previous DENV exposure among the participants and immunity against ZIKV or CHIKV – because ZIKV/CHIKV-immunity was an exclusion criterion for analysis of the ZIKV/CHIKV travel-acquired infections – we calculated the prevalence of previous DENV, ZIKV or CHIKV infection and the corresponding 95% confidence intervals (CI). Logistic regression analysis was used to examine determinants of previous infection. Variables with a p value < 0.1 in univariable analysis were included in the multivariable model and retrained in the final model using a backwards stepwise approach. A p value < 0.05 was considered statistically significant. Data were analysed using STATA versions 15.1 (StataCorp LLC, Texas, United States).

## Results

### Study population

Overall, 554 Dutch travellers, of whom 169 were VFR travellers, intended to participate. Of the total, 73 (13%) were excluded (Figure 1). Included in the Dutch tourist travellers were eight participants born in non-endemic countries other than the Netherlands. The resulting 481 participants consisted of 137 VFR and 344 tourist travellers. The overall median age was 48 years (interquartile range (IQR): 31–59), 177 (37%) were male, and the median travel duration was 22 days (IQR: 15–29). More than half of the participants (273, 57%) had no record of previous flavivirus vaccinations; 112 (23%) had received one and 96 (20%) had received two or more previous flavivirus vaccinations. The median interval between travel return and post-travel blood donation was 19 days (IQR: 16–23). The median age of the VFR travellers was 57 years (IQR: 49–62), 41 (30%) were male, the median travel duration was 29 days (IQR: 22–39), 51 (37% migrated before 1975 to the Netherlands and 64 (47%) lived 15–24 years in Suriname before migrating. the median age of the tourist travellers was 39 years (IQR: 27–55), 136 (40%) were male and the median travel duration was 19 days (IQR: 15–24).

### Travel characteristics

Of the 481 participants, 192 (40%) kept their travel diary daily, 125 (26%) retrospectively, and 148 (31%) kept it during travel but not in the 2 weeks post travel, for which information was obtained retrospectively. For 16 (3%) participants, this post-travel information was missing.

Overall, 275 pm were spent in Paramaribo, 64 pm in the coastal areas, 55 pm in inland areas and 8 pm in other countries (predominantly in neighbouring Guyana or French Guyana, or in the Dutch Antilles).

In total, 171 of 481 participants reported one or more symptoms which are frequently reported with dengue at least once. Headache was reported most often by these 171 participants (n = 341), followed by rash (n = 324), diarrhoea ≥ 3 times/day (n = 215), arthralgia (n = 182), muscle ache (n = 154), fever (n = 76), retro-orbital pain (n = 61) and vomiting (n = 54).

### Travel**-**acquired infections

**Figure 2:**
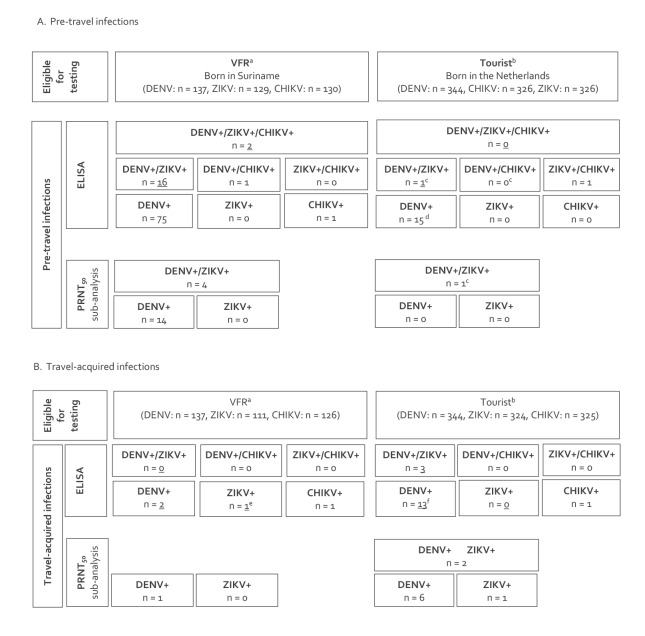
Serological results of pre-travel and travel-acquired dengue, Zika and chikungunya virus infections among Dutch travellers to Suriname, prospective study, the Netherlands, 2014–2017 (n = 481)

During travel, four VFR travellers acquired four infections: two had a DENV infection (one primary and one secondary, attack rate (AR)_DENV_: 1.5), one seroconverted for ZIKV (AR_ZIKV_: 0.9) and one for CHIKV (AR_CHIKV_: 0.8). In total, 20 infections were detected in 17 tourists: 16 with DENV (15 primary and one secondary, AR_DENV_: 4.7), three with ZIKV (AR_ZIKV_: 0.9) and one with CHIKV (AR_CHIKV_: 0.3). Of these tourists, three seroconverted for both DENV and ZIKV (Figure 2). In the Supplement (part 3), we provide additional information about the occurrence of these travel-acquired infections on a timeline.

+: positive test result; CHIKV: chikungunya virus; DENV: dengue virus; ELISA: enzyme-linked immunosorbent assay; PRNT50:  plaque-reduction neutralisation test (≥ 50% neutralisation); VFR: visiting friends and relatives; ZIKV: Zika virus.

^a^ Dutch travellers visiting friends and relatives in Suriname.

^b^ Dutch tourist travellers who visited Suriname (including 65 second-generation migrants).

^c^ Including one second-generation migrant.

^d^ Including seven second-generation migrants.

^e^ One travel-acquired infection was non-confirmed due to ZIKV-PRNT50 activity in pre-travel sample.

^f^ Two travel-acquired infections were non-confirmed due to DENV-PRNT50 activity in pre-travel sample.

The figure includes sub-analysis presenting DENV/ZIKV-PRNT50 results for selected participants. Underlined numbers indicate participants who were eligible for PRNT50 sub-analysis. As various numbers and combinations of pre-travel or travel-acquired infections were found, the results are grouped according to positive test results.

The overall IR were IR_DENV_ = 47.0 (95% CI: 29.6–74.6), IR_ZIKV_ = 11.6 (95% CI: 4.4–31.0) and IR_CHIKV_ = 5.6 (95% CI: 1.4–22.2) per 1,000 pm of travel. The IR for VFR travellers were IR_DENV_ = 13.6 (95% CI: 3.4–54.5), IR_ZIKV_ = 8.5 (95% CI: 1.2–60.5) and IR_CHIKV_ = 7.6 (95% CI: 1.1–54.1), and those for tourist travellers were IR_DENV_ = 67.8 (95% CI: 41.5–110.7), IR_ZIKV_ = 13.3 (95% CI: 4.3–41.1) and IR_CHIKV_ = 4.4 (95% CI: 0.6–31.0) per 1,000 pm of travel (Table 1, Figure 2). 

**Table 1:** Serologically determined travel-acquired dengue virus infection among Dutch VFR and tourist travellers to Suriname, prospective study during the primary introduction in Suriname of CHIKV and ZIKV, the Netherlands, 2014–2017 (n = 481)

Characteristics	Total			ELISA-determined travel-acquired DENV infections			PRNT_50_-confirmed travel-acquired DENV infections		Univariable analysis of PRNT_50_-confirmed DENV infections		
	n	%	pm	n	IR/1,000 pm	pm	n	IR/1,000 pm	IRR	95% CI	p value
Number of participants	481	100	383	18	47.0	386	9	23.3	NA		
Sex											
Male	177	37	141	3	21.3	141	2	14.2	1	Reference	0.352
Female	304	63	242	15	62.1	245	7	28.6	2.0	0.4–9.7	
Age in years											
≤ 35	160	33	115	7	61.0	116	4	34.4	1	Reference	0.634
36–55	161	33	125	5	40.1	126	2	15.9	0.5	0.09–2.5	
≥ 56	160	33	143	6	41.8	144	3	20.8	0.6	0.1–2.7	
Type of traveller											
Tourist (born in the Netherlands)	344	72	236	16	67.8	239	8	33.5	1	Reference	0.068
VFR (born in Suriname)	137	28	147	2	13.6	147	1	6.8	0.2	0.03–1.6	
Additional flavivirus vaccinations^a^											
0	229	48	197	7	35.6	198	3	15.1	1	Reference	0.277
1	252	52	186	11	59.1	188	6	31.9	2.1	0.5–8.4	
Visited areas											
Paramaribo only	67	14	62	2	32.2	63	1	16.0	1	Reference	0.665
Paramaribo and/or other areas	414	86	321	16	49.9	324	8	24.7	1.5	0.2–12.4	
Usage of DEET, % of total travel time											
< 25	113	23	118	3	25.4	118	3	25.4	1	Reference	0.182
25–49	55	11	39	1	25.8	40	0	0	NA		
50–74	58	12	49	1	20.5	49	0	0	NA		
≥ 75	255	53	177	13	73.5	179	6	33.5	1.3	0.3–5.3	
Year of (midpoint of) travel											
2014	142	30	119	1	8.4	119	1	8.4	1	Reference	0.222
2015	120	25	90	5	55.8	90	2	22.1	2.6	0.2–29	
2016	128	27	95	11	115.6	98	5	51.1	6.1	0.7–52	
2017	91	19	79	1	12.7	79	1	12.7	1.5	0.1–24	

CI: confidence interval; DEET: N,N-diethyl-meta-toluamide; DENV: dengue virus; ELISA: enzyme-linked immunosorbent assay; IR: incidence rate; IRR: incidence rate ratio; pm: person-months; NA: not applicable; PRNT50:  plaque-reduction neutralisation test (≥ 50% neutralisation); VFR: visiting friends and relatives.

^a^ Flavivirus vaccinations include yellow fever, tick-borne encephalitis and Japanese encephalitis vaccinations received during or after the pre-travel blood donation.

Of all 21 participants (four VFR and 17 tourists) with travel-acquired infections, 16 were female, the median age was 50 years (range: 19–70 years) and the median travel duration was 20 days (range: 10–89 days). Of the 19 participants with a DENV and/or ZIKV infection, seven reported at least one symptom during the study period, of whom only three reported fever (Table 2). Both participants with a CHIKV infection reported multiple symptoms including arthralgia (Table 2). Poisson regression analysis for serological determined travel-acquired DENV is described in part 4 of the Supplement. Regression analyses were not performed for ZIKV and CHIKV due to the small numbers. Sensitivity analyses, restricting the exposure period to the outbreak period, yielded an IR_ZIKV_ of 85.8 (95% CI: 27.7–266.1; n = 60) and an IR_CHIKV_ of 18.7 (95% CI: 4.7–74.6; n = 134) per 1,000 pm.

**Table 2:** Characteristics of Dutch VFR and tourist travellers to Suriname with a serologically determined travel-acquired dengue, chikungunya and/or Zika virus infection, including PRNT50 confirmation results, the Netherlands, 2014–2017 (n = 21)

Age group	Sex	Type of traveller	Flavivirus vaccination^a^		Dengue virus						Zika virus						Chikungunya virus		Year of departure	Travel time (days)	Destinations	Reported symptoms
			Pre-travel	Additional travel-related	ELISA (RU/mL)				PRNT_50_		ELISA (RU/mL)				PRNT_50_		ELISA (RU/mL)					
					Pre-travel IgG	Previous infection	Post-travel IgG	Travel-acquired infection	Previous infection	Travel-acquired infection	Pre-travel IgG	Previous infection	Post-travel IgG	Travel-acquired infection	Previous infection	Travel-acquired infection	Previous infection	Travel-acquired infection				
40–49	F	Tourist	0	1	36	**Yes**	177	**Yes**	**Yes**	**Yes**		No	4	No	No	No	No	No	2014	15	P'bo	Muscle ache
30–39	M	Tourist	0	1	ND	No	4	No	ND		ND	No	1	No	ND		No	**Yes**	2014	18	P'bo, coast, inland	Fever, headache, muscle ache, arthralgia, vomiting, rash
50–59	F	VFR	0	0	ND	No	7	No	ND		ND	No	1	No	ND		No	**Yes**	2014	32	P'bo, coast, inland	Headache, arthralgia, rash
60–69	F	Tourist	2	0	6	No	27	**Yes**	No	No	ND	No	< 2	No	No	No	No	No	2015	15	P'bo, coast, inland	None
18–29	F	Tourist	0	1	5	No	87	**Yes**	No	**Yes**	5	No	69	**Yes**	No	**Yes**	No	No	2015	89	P'bo	Arthralgia, rash
60–69	F	Tourist	0	1	2	No	23	**Yes**	No	No	ND	No	15	No	No	No	No	No	2015	17	P'bo, coast, inland	Fever, headache, muscle ache, nose bleeding
50–59	F	Tourist	1	1	< 2	No	65	**Yes**	No	No	ND	No	7	No	No	No	No	No	2015	17	P'bo, coast, inland	Fever
60–69	F	Tourist	2	0	< 2	No	114	**Yes**	No	**Yes**	< 2	No	129	**Yes**	No	**Yes**	No	No	2015	31	P'bo, coast, inland	Fever, headache, arthralgia, rash
18–29	F	Tourist	0	1	3	No	132	**Yes**	No	No	2	No	106	**Yes**	No	**Yes**	No	No	2015	19	P'bo, inland	None
50–59	F	VFR	1	0	14	No	51	**Yes**	No	No	ND	No	2	No	No	No	No	No	2016	33	P'bo, coast	Arthralgia
18–29	F	Tourist	0	1	20	No	164	**Yes**	No	**Yes**	ND	No	< 2	No	No	No	No	No	2016	20	P'bo, coast, inland	None
18–29	M	Tourist	2	0	8	No	34	**Yes**	No	No	ND	No	3	No	No	No	No	No	2016	24	P'bo, coast, inland	None
50–59	F	Tourist	2	0	13	No	87	**Yes**	No	**Yes**	ND	No	6	No	No	No	No	No	2016	19	P'bo, inland, Dutch Antilles (10 days)	None
18–29	F	Tourist	0	1	8	No	50	**Yes**	Brd	No	ND	No	2	No	No	No	No	No	2016	53	P'bo, coast, inland	Headache, diarrhoea, rash
30–39	M	VFR	0	1	153	**Yes**	179	No	**Yes**	No	15	No	39	**Yes**	**Yes**	No	No	No	2016	21	P'bo, inland	None
18–29	F	VFR	0	1	31	**Yes**	185	**Yes**	Yes	**Yes**	ND	No	6	No	No	No	No	No	2016	30	P'bo, coast	None
30–39	F	Tourist	0	1	7	No	39	**Yes**	No	**Yes**	ND	No	2	No	No	No	No	No	2016	12	P'bo, inland	None
50–59	F	Tourist	0	1	6	No	45	**Yes**	**Yes**	No	ND	No	2	No	No	No	No	No	2016	24	P'bo, Guyana (3 days)	None
50–59	F	Tourist	2	0	14	No	29	**Yes**	**Yes**	No	ND	No	7	No	**Yes**	No	No	No	2016	15	P'bo, inland	None
60–69	M	Tourist	0	1	< 2	No	44	**Yes**	No	**Yes**	ND	No	3	No	No	No	No	No	2016	10	P'bo, coast, inland	None
70–79	M	Tourist	1	0	9	No	180	**Yes**	No	**Yes**	ND	No	5	No	No	No	No	No	2017	22	P'bo, coast	None

Brd: borderline; ELISA: enzyme-linked immunosorbent assay; F: female; M: male; IgG: immunoglobulin G; ND: not done; P’bo: Paramaribo; PRNT50: plaque-reduction neutralisation test (≥ 50% neutralisation); RU: relative units; VFR: traveller visiting friends and relatives.

^a^ Yellow fever, tick-borne encephalitis and Japanese encephalitis.

DENV and ZIKV PRNT50 was performed in participants with an ELISA-confirmed travel-acquired dengue and/or Zika virus infection.

### PRNT50-confirmed travel-acquired infections

In VFR travellers, one of two DENV (AR_DENV_: 0.7) serology-based travel-acquired infections were PRNT50-confirmed; the one ZIKV (AR_ZIKV_: 0) was not PRNT50-confirmed (Figure 2, Table 2). In tourist travellers, eight of 16 DENV (AR_DENV_: 2.3) and all three ZIKV travel-acquired infections were PRNT50-confirmed (Figure 2, Table 2). Of the three tourist travellers who were serologically positive for a travel-acquired infection with both DENV and ZIKV, two were PRNT50-confirmed for both and the third was confirmed for travel-acquired ZIKV infection only (Figure 2).

Based on PRNT50-confirmed travel-acquired infections, IR_DENV_ was 23.3 (95% CI: 12.1–44.8) and IR_ZIKV_ 8.4 (95% CI: 2.7–26.1) per 1,000 pm of travel. The IR for VFR travellers were IR_DENV_ 6.8 (95% CI: 1.0–48.2) and IR_ZIKV_ 0 (95% CI: 0–0.024) and for tourist travellers, IR_DENV_ 33.5 (95% CI: 16.8–67.0) and IR_ZIKV_ 13.2 (95% CI: 4.3–41.0) per 1,000 pm of travel. In univariable Poisson regression analysis, tourist travel was associated with PRNT50-confirmed travel-acquired DENV infection (IRR = 4.9; 95% CI: 0.6–39.4). Although the effect was not statistically significant, 2016 was the calendar year with the highest overall (VFR and tourist) IR_DENV_ of 51.1 (95% CI: 21.3–122.8) per 1,000 pm (IRR = 6.1; 95% CI: 0.7–52.2), compared with 2014, 2015 and 2017.

Sensitivity analysis based on the PRNT50 test results yielded an overall IR_ZIKV_ of 83 (95% CI: 26.9–258.3; n = 61) per 1,000 pm during the outbreak period for travel-acquired ZIKV infection.

### Pre-travel infections

Of the VFR travellers, 95 had serological evidence of previous infection: 94 of 137 (69%; 95% CI: 60–76) DENV, 18 of 129 (14%; 95% CI: 8.5–21) ZIKV, and four of 130 (3%; 95% CI: 0.9–7.7) CHIKV. In contrast, 17 tourist travellers had serological evidence of a previous infection: 16 of 344 (5%; 95% CI: 2.7–7.4) DENV, two of 326 (0.6%; 95% CI: 0.1–2.1) ZIKV and one of 326 (0.3%; 95% CI: 0.1–1.7) CHIKV (Table 3 and Figure 2). Nineteen participants (18 VFR, one tourist) tested positive for both a previous DENV and ZIKV infection, of whom two (both VFR) also tested positive for previous CHIKV infection. One VFR tested positive for both previous DENV and CHIKV and one tourist for both ZIKV and CHIKV infections (Figure 2). Of the 460 participants for whom this information was available, 13 of 460 reported having had a previous DENV infection, of whom nine were serologically confirmed. Of the 19 participants (18 VFR, one tourist) who tested positive for DENV as well as ZIKV before travel, only five were PRNT50-confirmed for a previous infection with both DENV and ZIKV. The remaining 14 (all VFR) were confirmed only for a previous DENV infection (Figure 2). Characteristics including ELISA and PRNT test results of these 19 participants care provided in part 5 of the Supplement.

**Table 3:** Characteristics and serological evidence of previous infection in Dutch VFR and tourist travellers to Suriname attending a Dutch travel health clinic for pre-travel advice and participating in a prospective study of travel-acquired DENV, ZIKV and CHIKV infections, the Netherlands, March 2014-October 2017 (n = 481)

Characteristics	Total	%	Previous DENV infection		Univariable analysis		
			n	%^c^	OR	95% CI	p value
Participants	481	100	110	23			
Sex							
Male	177	37	35	20	1	Reference	0.214
Female	304	63	75	25	1.3	0.8–2.1	
Age in years							
≤ 35	160	33	10	6	1	Reference	< 0.001
36–55	161	33	40	25	5.0	2.4–10.3	
≥ 56	160	33	60	38	9.0	4.4–18.4	
Type of traveller							
Tourist (born in the Netherlands)	344	72	16	5	1	Reference	< 0.001
VFR (born in Suriname)	137	28	94	69	45	24.2–83.1	
Total of pre-travel flavivirus vaccinations^a^							
0	273	57	53	19	1	Reference	0.039
≥ 1	208	43	57	27	1.6	1.0–2.4	
Calendar year of migration (VFR travellers only)^b^							
≤ 1974	51	37	29	57	1	Reference	0.010
1975–1981	37	27	23	62	1.2	0.5–3.0	
≥ 1982	44	32	37	84	4.0	1.5–10.7	
Data missing	5	4	NA		NA		
Years lived in Suriname before migration (VFR travellers only)^b^							
≤ 15	34	25	6	18	1	Reference	< 0.001
15–24	64	47	54	84	25	8.3–76.5	
≥ 25	34	25	29	85	27	7.4–98.9	
Data missing	5	4	NA		NA		

CHIKV: chikungunya virus; CI: confidence interval; DENV: dengue virus; NA: not applicable; OR: odds ratio; VFR: visiting friends and relatives; ZIKV: Zika virus.

^a^ Flavivirus vaccinations include yellow fever, tick-borne encephalitis and Japanese encephalitis vaccinations.

^b^ Subgroup analysis: not applicable for multivariable analysis.

^c^ These numbers reflect the percentages of previous infections among the total number of participants of the corresponding category.

In univariable logistic regression analysis of the serology-based results, previous infections were associated with VFR travelling (DENV, ZIKV, CHIKV), older age (DENV, ZIKV) and a history of at least one flavivirus vaccination (DENV) (Table 3); the logistic regression analysis to previous ZIKV and CHIKV are appended in the Supplement, part 6. In the multivariable model, only VFR travelling remained significantly associated with a previous DENV infection (OR: 38; 95% CI: 20–76).

## Discussion

This prospective study among Dutch tourist and VFR travellers to Suriname found a considerable incidence of travel-acquired DENV infections which was five times higher in tourist than in VFR travellers, although the 95% CI overlapped. This finding is in contrast with studies of other travel-related illnesses which found a higher incidence of travel-acquired infections in VFR travellers [10-12]. Although not statistically significant, travelling in 2016 was also associated with travel-acquired DENV infection.

The overall IR of travel-acquired DENV infection based on 47.0 per 1,000 pm is within the range of previous reported serology-based prospective studies among travellers (6.7–58.7 per 1,000 pm) [7,20-25]. Of these studies, only one used PRNT50 in a selection of participants; as in our study, it could not confirm all serology-based DENV infections. This suggests that serology-based DENV infection rates are likely to overestimate the true incidence.

For DENV, we found the highest overall IR in 2016. Peaks of DENV infections occur every 3 to 4 years in dengue-endemic areas in South America [26]. From 2001 through 2012, peaks in DENV infections in Suriname were recorded in 2009 and 2012, and our finding of a peak in 2016 appears to correspond with this cycle [27]. Surprisingly, the Pan American Health Organisation reported only six DENV cases in Suriname in 2016, and this total was lower than in the preceding 2 years [28]. Although under-reporting is possible, an unexpectedly low number of DENV cases was likewise reported in other American countries following the large ZIKV epidemics in 2015 and 2016, possibly due to temporary cross-protection by recent ZIKV infections [29,30].

For ZIKV and CHIKV, the incidence was highest during our estimated outbreak periods in Suriname. No CHIKV infections were found outside the outbreak period (see the arrows on the timeline in Supplement part 3), an observation comparable to those of a previous study [31]. We identified only one other small prospective study which estimated ZIKV infection incidence among travellers; it reported a somewhat higher incidence of 17% per pm (170 per 1,000 pm) among 49 Belgian travellers to South America in 2016 [32].

Many European countries have close ties with populations from former colonies, or large groups of migrants from other sub-tropical countries that are endemic for DENV and other arboviruses. With increasing travel, these arboviral infections can cause a large and increasing disease burden in returning travellers. The arthropod vector for DENV, ZIKV and CHIKV is expanding rapidly in continental Europe and in the past 15 years, multiple local dengue and chikungunya outbreaks have been reported after virus introduction by a viraemic traveller [33-41]. More insight in travel risk groups and determinants for disease is needed, both for travel health consultations and for arbovirus preparedness and control in Europe.

Our study was designed to investigate the risks and predictors of DENV infection based on serology. After the introduction of ZIKV and CHIKV in Suriname, however, we expanded the study to include these two viruses because of potential serological cross-reactions between DENV and ZIKV viruses and our interest in the risks of these two additional mosquito-borne infections for travellers. To study potential cross-reactions of DENV and ZIKV antibodies, we performed additional DENV and ZIKV PRNT50 confirmation tests, using selected samples due to budget limitations.

Cross-reaction probably contributed to a substantial overestimation of the prevalence of anti-ZIKV antibodies in VFR travellers, as most pre-existing ZIKV antibodies in VFR with both anti-ZIKV and anti-DENV antibodies were not confirmed in PRNT50. All non-confirmed ZIKV cases in this group had very high titres of anti-DENV antibodies, which probably caused cross-reactions in the anti-ZIKV IgG ELISA test (according to the test results appended in the Supplement, part 5). The confirmation of false positivity in these participants was also expected because it was unlikely that they had travelled since the ZIKV introduction in Suriname in 2015. The PRNT50 results of the five participants with confirmed pre-existing DENV and ZIKV should therefore also be interpreted with care. Another study also found cross-neutralising antibody responses in focus reduction neutralisation tests (FRNT50), however cross-reacting ZIKV antibody responses showed lower neutralisation activity compared with the antibodies against the infecting DENV serotype [42]. Perhaps a stricter cut-off, such as PRNT90, would be more accurate in participants who demonstrate both DENV and ZIKV antibodies in serological tests.

The incidence of travel-acquired DENV infections may have been overestimated, but possibly for other reasons than cross-reactivity, as eight of nine travellers with a non-confirmed DENV infection in PRNT50 had no ZIKV co-infection, and four of nine did not receive any additional flavivirus-vaccines. Although PRNT50 is still considered the most reliable test to confirm seroconversion, PCR in the viraemic phase of disease remains the gold standard. Further research is necessary to gain more insight into the associations between clinical symptoms, serological test results and PCR-confirmed DENV and ZIKV infections.

After the Zika epidemic in the Americas, a remarkable temporary decline in DENV infections was seen across the Americas, for which temporarily cross-protecting ZIKV antibodies have been hypothesised as a possible reason [29]. While cross-reactions of antibodies can overestimate incidences based on serology, cross-protection of anti-DENV and anti-ZIKV antibodies can lead to underestimations of infection risks in non-immune persons. Cross-protection against congenital ZIKA syndrome due to DENV antibodies was found in north-eastern Brazil [43,44]. Although (intermediate antibody levels from) prior infections have also been associated with more severe disease, cross-protection could perhaps be a reason why none of our travellers with high titres of DENV antibodies contracted a travel-acquired ZIKV infection and why none with a travel-acquired DENV infection had evidence of pre-existing anti-ZIKV antibodies [45].

A further argument for the cross-protection hypothesis is that all 19 participants with pre-existing antibodies to both DENV and ZIKV had high serological anti-DENV titres (DENV IgG titres ≥ 125 relative units/mL), and the 17 with complete PRNT50 results all had neutralising activity of ≥ 90% against at least two DENV serotypes in PRNT50 (see the extra material in part 5 of the Supplement). It is likely that these 19 travellers, all but one VFR, had a past secondary DENV infection and therefore not at risk for following DENV infections anymore. These VFR travellers, and probably other participants with high pre-existing anti-DENV antibody titres that we could not retest, contributed to the low incidence of DENV infections among VFR travellers.

Finally, it is noteworthy that four of five previous CHIKV infections were among VFR travellers, although CHIKV was introduced only recently. Other alphaviruses such as Mayaro virus may have cross-reacted with the CHIKV test and caused false-positive results [46,47].

Our study has some limitations. Firstly, as participants were recruited at a travel vaccination clinic, they may have had a higher health awareness than those not seeking pre-travel health advice; thus, our findings may not be generalisable to all travellers to Suriname. Secondly, as risk of infection can differ between first- and second-generation migrants, these two groups are preferably analysed separately, but this analysis was not part of our study. Thirdly, some data such as pre-study travel history were incomplete and could therefore not be used in our analyses. Fourthly, no risk factors could be identified for ZIKV or CHIKV infection due to our small numbers. Finally, the default answers regarding symptoms and use of DEET in the diary were ‘no’ or ‘not used’, respectively. Therefore, symptoms or usage of DEET could have been under-reported if participants forgot to tick the box in the diary, making potential associations more difficult to demonstrate.

## Conclusions

Dutch tourist and VFR travellers to Suriname, especially tourist travellers, run a substantial risk of contracting DENV, and since the introduction of ZIKV and CHIKV, they run a considerable risk of ZIKV and CHIKV infections during outbreaks as well. Cross-reacting anti-DENV antibodies probably contributed to an overestimation of the pre-travel ZIKV prevalence and cross-reaction may also have contributed to overestimation of the travel-acquired incidence of DENV infections. Conversely, high titres of pre-travel DENV antibodies potentially protect against other flavivirus infections. As the habitat of the vectors for DENV, ZIKV and CHIKV has expanded in recent years, expanded epidemiological knowledge of these arboviruses will be necessary both for travel health advice and for arbovirus control in Europe. Future studies into the incidence of flavivirus infections should consider the use of diagnostic tools during travel such as dried blood spots (travellers collecting their own blood sample using a finger prick) that allow for PCR testing after travel. This will offer additional information about the infecting virus and serotype and lead to more reliable incidence estimates in travellers. 

## Supplementary Data

417000 Supplement
