# Supplementary material for: Dengue, chikungunya and Zika virus infections among Dutch travellers to Suriname: a prospective study during the introduction of chikungunya and Zika virus, 2014 to 2017
Source: Euro Surveill. 2023 Jan 12;28(2):2200344. doi: 10.2807/1560-7917.ES.2023.28.2.2200344 (PMC9837856; doi:10.2807/1560-7917.ES.2023.28.2.2200344)
Supplement: Supplement [file 22-00344_OVERBOSCH_SupplementaryMaterial.pdf]

Supplementary files (1-6)

This supplementary material is hosted by Eurosurveillance as supporting information alongside the article Incidence of dengue, chikungunya and Zika virus infections among Dutch travellers to Suriname: a prospective study among tourists and travellers visiting friends and relatives during the recent introduction of chikungunya and Zika virus, on behalf of the authors, who remain responsible for the accuracy and appropriateness of the content. The same standards for ethics, copyright, attributions and permissions as for the article apply. Supplements are not edited by Eurosurveillance and the journal is not responsible for the maintenance of any links or email addresses provided therein.

Supplementary 1

The questionnaire participants filled out daily in their travel diary (original travel diary in Dutch). The data was collected for each day of travel, including 14 days post-travel.

Week no.	Monday	Tuesday	Wednesday	Thursday	Friday	Saturday	Sunday
Date							
**General information**	Tick all boxes and/or answer		the questions which apply to you				
Stay in Suriname:							
- Paramaribo	❑	❑	❑	❑	❑	❑	❑
- coastal area	❑	❑	❑	❑	❑	❑	❑
- inlands	❑	❑	❑	❑	❑	❑	❑
Stay in the Netherlands:	❑	❑	❑	❑	❑	❑	❑
Stay in other country, namely:							
Used DEET?	❑	❑	❑	❑	❑	❑	❑
**Symptoms**	Tick all boxes and/or answer		the questions which apply to you				
Fever	❑	❑	❑	❑	❑	❑	❑
If yes, body temperature:	°C	°C	°C	°C	°C	°C	°C
How did you measure? (rectally/axillary/orally)							
Headache	❑	❑	❑	❑	❑	❑	❑
Retro-orbital pain	❑	❑	❑	❑	❑	❑	❑
Muscle ache (unrelated to physical activity)	❑	❑	❑	❑	❑	❑	❑
Arthralgia	❑	❑	❑	❑	❑	❑	❑
Vomiting	❑	❑	❑	❑	❑	❑	❑
Diarrhoea ≥ 3x/day	❑	❑	❑	❑	❑	❑	❑
Rash	❑	❑	❑	❑	❑	❑	❑
Other symptoms, namely:*							
**Needed treatment?**	Tick all boxes and/or answer		the questions which apply to you				
Measures taken by itself?	❑	❑	❑	❑	❑	❑	❑
- Medication:*							
- Other, namely:*							
Did you consult a physician?	❑	❑	❑	❑	❑	❑	❑
- If yes, why?*							
Blood donation collected?	❑	❑	❑	❑	❑	❑	❑
Diagnosis:*							
Treatment initiated by the physician?	❑	❑	❑	❑	❑	❑	❑
- Medication:*							
- Hospitalised	❑	❑	❑	❑	❑	❑	❑
- other, namely:*							

* = if necessary, continue answer under notes

Notes:

....

....

Supplementary 2

Procedures used for the in-house (Amsterdam University Medical Centers) Plague Reduction Neutralization Test$_{50}$ against Dengue virus serotypes 1-4 and Zika virus.

An in-house Plaque Reduction Neutralization Test (PRNT$_{50}$) was performed to determine the virus type-specific neutralizing antibody response against dengue virus (DENV) serotypes 1-4 and Zika virus (ZIKV). Samples of selected participants were tested in pair. Participants were selected for PRNT$_{50}$ if: 1) the pre-travel anti-DENV and anti-ZIKV Enzyme Linked ImmunoSorbent Assay (ELISA) Immunoglobulin (Ig)G yielded a positive test result (n=19), or 2) a travel-acquired DENV and/or ZIKV infection was determined based on the pre- and post-travel anti-DENV and anti-ZIKV ELISA IgG test results (n=19).

The serum was heat-inactivated at 56˚C in a water bath for 30 minutes and 1:10 diluted. Next, 200 µL serum and a 200 µL virus control (Eagle's Minimal Essential Medium 2% Fetal Calf Serum) was mixed with an equal volume of each reference virus (DENV serotype 1-4 or ZIKV). The virus-serum mixture was then incubated at 37°C in a 5% CO_2 incubator for 1 hour. The virus-serum mixture was inoculated on VERO cell line in wells of a 6-well plate and incubated at 37°C in a 5% CO_2 incubator for 1 hour.
After removal of excess virus-serum mixture, 1ml overlay medium was added to each well, and this was allowed to solidify for 15 minutes at room temperature. Plates were incubated at 37°C in a 5% CO_2 incubator for 5 days. For fixation after incubation 10% formaldehyde was added for 20 minutes at room temperature.
After removal of the overlayer, plates were washed 3 times. One ml Cristall Violett was added for incubation of 30 minutes at room temperature. At last, the plates were washed with water 5 times and air-dried at room temperature. The plaques were now ready for counting.

A positive control (virus without sera) were included for each assay. In addition, positive serum controls with high-titer convalescent sera against four dengue serotypes or Zika virus were included as well.

The percentage of plaques counted in test sera was compared with the number of plaques from the virus control (without sera):
<50% inhibition compare to the positive control was considered negative
50% inhibition compare to the positive control was considered borderline
>50% inhibition compare to the positive control was considered positive

Supplementary 3

Time-line of our prospective study from 2014-2017 (a-d) among Dutch VFR and tourist travellers to Suriname, representing all participants. Travellers who contracted a serologically determined travel-acquired DENV, CHIKV and/or ZIKV infection are marked.

Each line represents the travel period of a participant. Dark blue line: no travel-acquired infection, red: travel-acquired DENV infection, orange: travel-acquired CHIKV infection, purple: travel-acquired ZIKV infection, brown: travel-acquired DENV and ZIKV infections

Orange arrow: first reported chikungunya case in Suriname*. Purple arrow: first reported Zika case in Suriname^.

a..

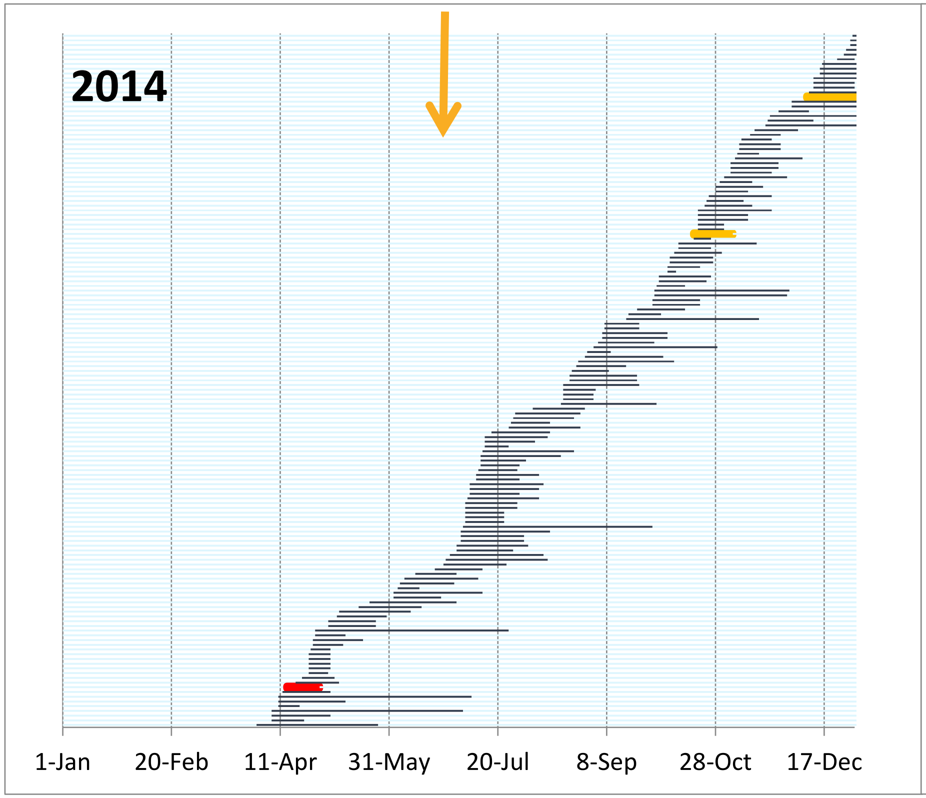

b.

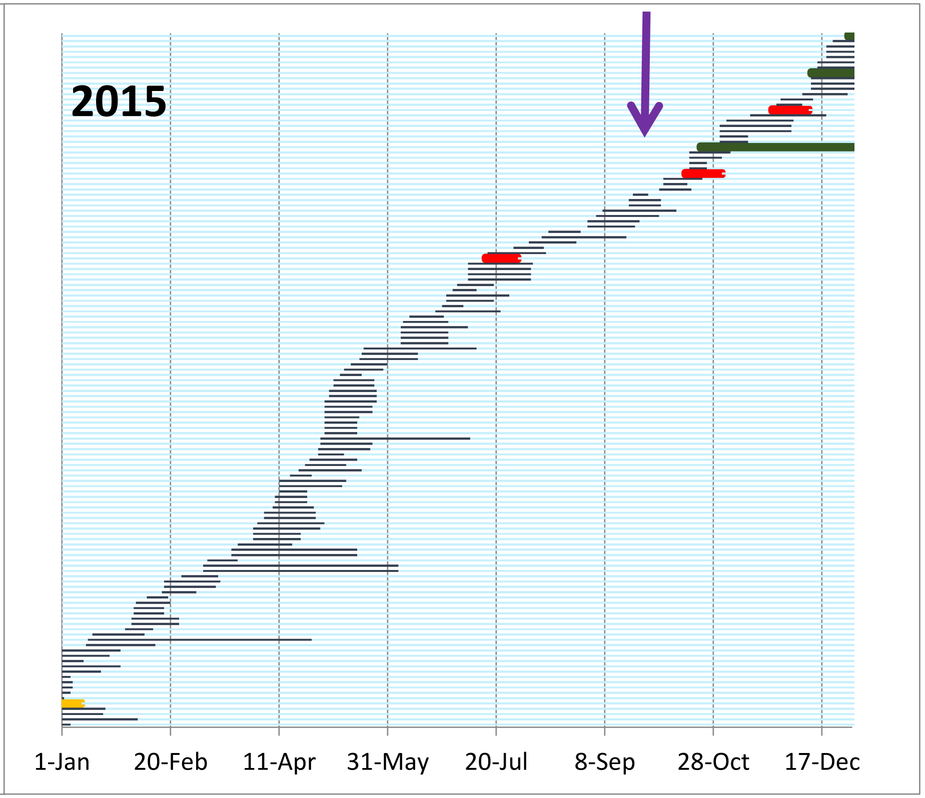

c.

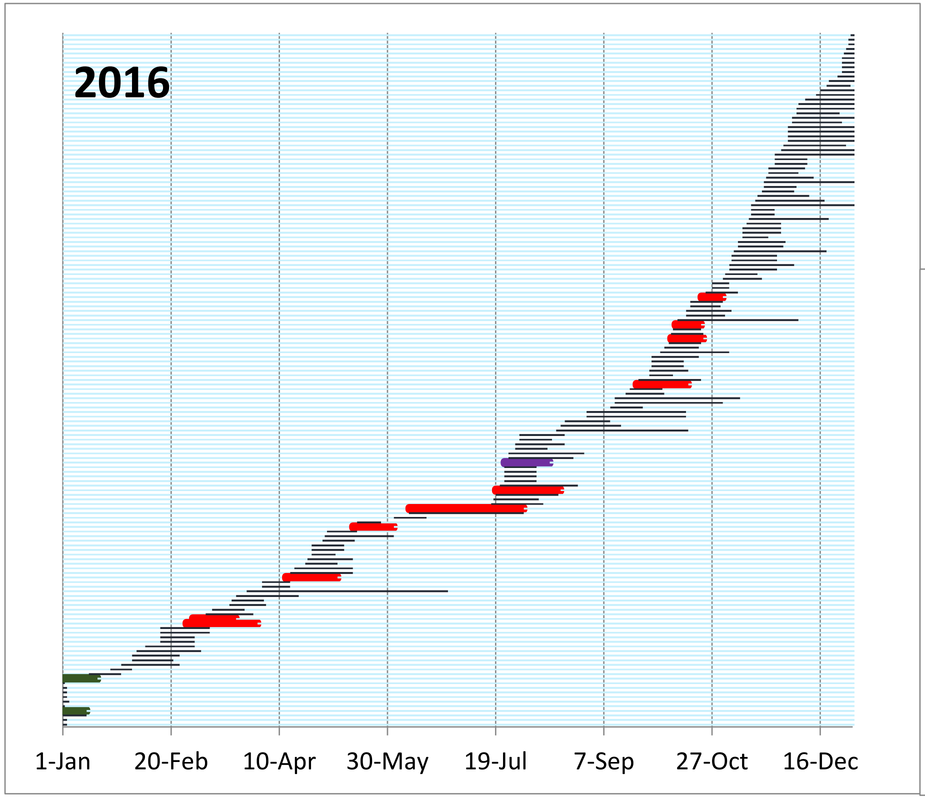

d.

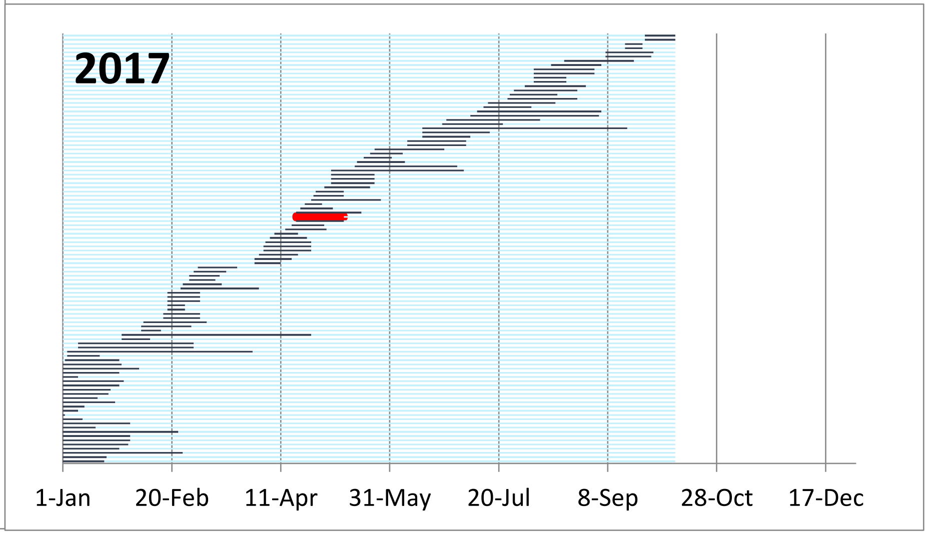

* Sensitivity analysis starting from the first reported CHIKV infection in Suriname onwards yielded a serology-based overall IR_{CHIKV} of 5.8 (96% CI: 1.5-23.3 per 1,000 pm of travel (n= 435 participants).

^ Sensitivity analysis starting from the first reported ZIKV infection in Suriname onwards yielded a serology-based overall IR_{ZIKV} of 23.4 (95% CI: 8.8-62.4) per 1,000 pm of travel (n=226 participants), and an overall IR_{ZIKV} of 16.8 (95% CI: 5.4-52.0) per 1,000 pm of travel based on PRNT50 test results (n=234).

Supplementary 4

Serologically determined travel-acquired DENV infection among Dutch VFR and tourist travellers to Suriname: a prospective study of travel-acquired DENV, ZIKV and CHIKV infections during the primary introduction of CHIKV and ZIKV in Suriname, 2014-2017 (n=481).

	Total			travel-acquired **DENV** infections		univariable analysis				multivariable analysis			
							95% CI				95% CI		
Characteristics	No.	%	person-months	No.	IR/1000 pm	IRR	lower	upper	p value	IRR	lower	upper	p value
no. Participants	481	100%	383	18	47.0								
Gender									0.059				0.037
Male	177	37%	141	3	21.3	1				1			
female	304	63%	242	15	62.1	2.9	0.8	10.1		3.2	0.9	11.2	
Age, y									0.720				
≤35	160	33%	115	7	61.0	1							
36-55	161	33%	125	5	40.1	0.7	0.2	2.1					
≥56	160	33%	143	6	41.8	0.7	0.2	2.0					
Type of traveler									0.009				0.005
Tourist (born in the Netherlands)	344	72%	236	16	67.8	1				1			
VFR (born in Suriname)	137	28%	147	2	13.6	0.2	0.04	0.9		0.2	0.04	0.8	
Additional flavivirus vaccinations^									0.286				
0	229	48%	197	7	35.6	1							
1	252	52%	186	11	59.1	1.7	0.6	4.3					
Visited areas									0.536				
Paramaribo only	67	14%	62	2	32.2	1							
Paramaribo and/or other areas	414	86%	321	16	49.9	1.6	0.4	6.7					
Usage of DEET, % of total travel time									0.168				
<25	113	23%	118	3	25.4	1							
25-49	55	11%	39	1	25.8	1.0	0.1	9.8					

50-74	58	12%	49	1	20.5	0.8	0.08	7.8					
≥75	255	53%	177	13	73.5	2.9	0.8	10.2					
Year of (midpoint of) travel									0.002				0.001
2014	142	30%	119	1	8.4	1				1			
2015	120	25%	90	5	55.8	6.7	0.8	57		7.2	0.8	62	
2016	128	27%	95	11	115.6	13.8	1.8	107		14.6	1.9	113	
2017	91	19%	79	1	12.7	1.5	0.1	24		1.7	0.1	27	

DENV= dengue virus

^=Flavivirus vaccinations include yellow fever, tick-borne encephalitis and Japanese encephalitis vaccinations received during or after the pre-travel blood donation.

Supplementary 5

Characteristics of Dutch VFR and tourist travellers to Suriname with serologically determined previous DENV and ZIKV infections, including PRNT50 confirmation results, 2014-2017 (n=19).

	agegroup	gender	type of traveler	years in Suriname before migration	total pre-travel FV vaccinations	pre-travel anti-DENV IgG RU/ml (ELISA)	result previous DENV inf (ELISA)	result previous DENV inf (PRNT50)	% neutralization DENV1-PRNT50	% neutralization DENV2-PRNT50	% neutralization DENV3-PRNT50	% neutralization DENV4-PRNT50	pre-travel anti-ZIKV IgG RU/ml (ELISA)	result previous ZIKV inf (ELISA)	result prev ZIKV inf (PRNT50)	% neutralization (ZIKV-PRNT50)	% neutralization (repeated ZIKV-PRNT50)	year of departure
1	50-59	F	VFR	>=25	1	143	P	P	76	100	96	84	42	P	N			2014
2	50-59	F	VFR	>=25	2	129	P	P	100	100	99	100	184	P	N			2014
3	60-69	M	VFR	15-24	0	133	P	P	100	100	91	76	41	P	N			2014
**4**	**40-49**	**M**	**VFR**	>=25	**1**	**150**	**P**	**P**	100	100	96	73	**73**	**P**	**P**	54		**2014**
**5**	**50-59**	**M**	**VFR**	15-24	**2**	**> 200**	**HP**	**P**	100	100	97	96	**52**	**P**	**P**	58		**2015**
6	50-59	M	VFR	15-24	2	156	P	P	100	100	97	96	44	P	N			2015
7	50-59	F	VFR	>=25	0	154	P	P	100	100	100	69	116	P	N			2015
8	60-69	F	VFR	>=25	2	125	P	P	100	100	96	63	46	P	N			2015
9	40-49	M	VFR	>=25	0	194	P	P	100	100	97	97	156	P	N			2015
10	80-89	F	VFR	15-24	1	154	P	P	100	97	99	100	74	P	N			2016
11	70-79	M	VFR	>=25	2	152	P	P	100	100	97	97	40	P	N			2016
12	60-69	F	VFR	15-24	2	140	P	P	na*	na*	87	na*	81	P	N			2016
13	60-69	F	VFR	>=25	0	177	P	P	91	100	88	86	49	P	N			2016
14	40-49	F	VFR	m	1	180	P	P	96	100	93	97	32	P	N			2016
**15**	60-69	M	VFR	>=25	0	189	P	P	100	100	96	83	49	P	N			2016
**16**	**40-49**	**F**	**tourist**	na	**0**	**> 200**	**HP**	**P**	100	100	99	94	**> 200**	**HP**	**P**	100		**2016**
17	60-69	F	VFR	15-24	1	161	P	P	na*	na*	96	na*	114	P	N			2017
18	**60-69**	**F**	**VFR**	15-24	**0**	**170**	**P**	**P**	100	100	100	97	**34**	**P**	**P**	H	100	**2017**
19	**50-59**	**F**	**VFR**	>=25	**1**	**190**	**P**	**P**	100	100	95	100	**80**	**P**	**P**	52	-32	**2017**

FV=flavivirus (yellow fever, tick-borne encephalitis and Japanese encephalitis) vaccinations
M=missing, N=negative, NA=not applicable, na*=% neutralization in post-travel sample: 100%,
P=positive (ELISA: ≥22 relative units/ml; PRNT50: >50%), HP=high positive (ELISA: >200 relative units/ml)
VFR=travelers visiting friends and relatives
In bold: participants with serologically and PRNT50-confirmed DENV and ZIKV infections.
prev DENV inf (ELISA)= serologically confirmed previous DENV infections using an anti-DENV IgG test (enzyme-linked immunosorbent assay)
prev DENV inf ($PRNT_{50}$)= $PRNT_{50}$-confirmed previous DENV infections among participants with a serologically confirmed previous DENV and ZIKV infections.
prev ZIKV inf (ELISA)= serologically confirmed previous ZIKV infections using an anti-ZIKV IgG test (enzyme-linked immunosorbent assay)
prev ZIKV inf ($PRNT_{50}$)= $PRNT_{50}$-(non)-confirmed previous ZIKV infections among participants with a serologically confirmed previous DENV and ZIKV infections.

Supplementary 6

A. Determinants of serologically determined previous ZIKV infection among Dutch VFR and tourist travellers to Suriname who attended a Dutch travel health clinic for pre-travel advice and participated in a prospective study of travel-acquired DENV, ZIKV and CHIKV infections, 2014-2017 (n=455).

Characteristics	Total, no	%	previous **ZIKV** inf No.	%	univariable analysis OR	95% CI upper	lower	p value
no. Participants	455	100	20	4.4%				
Gender								0.871
male	167	37	7	4.2%	1			
female	288	63	13	4.5%	1.1	0.4	2.8	
Age, y								0.013
≤55	303	67	8	2.6%	1			
≥56	152	33	12	7.9%	3.2	1.3	7.9	
Type of traveller								<0.001
Tourist (born in the Netherlands)	326	72	2	0.6%	1			
VFR (born in Suriname)	129	28	18	14%	26	6.0	115	
Total of pre-travel flavivirus vaccinations^								0.106
0	262	58	8	3.1%	1			
≥1	193	42	12	6.2%	2.1	0.8	5.3	
Year of migration (VFRs only) $								0.460
≤1974	48	37	5	10%	1			
1975-1981	35	27	4	11%	1.1	0.3	4.5	
≥1982	42	33	8	19%	2.0	0.6	6.7	
Data missing	4	3						
Years lived in Suriname pre-migration (VFRs only) $								
≤15	31	24	0	0	na			
15-24	60	47	7	12	na			
≥25	34	26	10	29	na			
Data missing	4	3						

VFR= visiting friends and relatives, ZIKV= Zika virus,
^=Flavivirus vaccinations include yellow fever, tick-borne encephalitis and Japanese encephalitis vaccinations.
$=subgroup analysis: not applicable for multivariable analysis

B. Determinants of serologically determined previous CHIKV infection among Dutch VFR and tourist travellers to Suriname who attended a Dutch travel health clinic for pre-travel advice and participated in a prospective study of travel-acquired DENV, ZIKV and CHIKV infections, 2014-2017 (n=456).

Characteristics	Total, no	%	previous **CHIKV** inf No.	previous **CHIKV** inf %	OR	univariable analysis 95% CI upper	univariable analysis 95% CI lower	p value
no. Participants	456	100	5	1.1				
Gender								0.417
male	167	37	1	0.60	ref			
female	289	63	4	1.4	2.3	0.26	21.0	
Age, y								0.221
≤55	304	67	2	0.66	ref			
≥56	152	33	3	2.0	3.0	0.50	18.4	
Type of traveller								0.016
Tourist (born in the Netherlands)	326	71	1	0.31	ref			
VFR (born in Suriname)	130	29	4	3.1	10.3	1.1	93.2	
Year of migration (VFRs only)								
≤1974	48	37	3	6.3	na			
1975-1981	36	28	1	2.8	na			
≥1982	42	32	0	0	na			
missing	4	3						
Years lived in Suriname before migration (VFRs only)								
≤15	32	25	1	3.1	na			
15-24	60	46	3	5.0	na			
≥25	34	26	0	0	na			
missing	4	3						

VFR= visiting friends and relatives, CHIKV= chikungunya virus,
^=Flavivirus vaccinations include yellow fever, tick-borne encephalitis and Japanese encephalitis vaccinations.
$=subgroup analysis: not applicable for multivariable analysis
